# Factors associated with baseline smoking self-efficacy among male Qatari residents enrolled in a quit smoking study

**DOI:** 10.1371/journal.pone.0263306

**Published:** 2022-01-27

**Authors:** Mohammed Al Thani, Vasiliki Leventakou, Angeliki Sofroniou, Hamza I. Butt, Iman A. Hakim, Cynthia Thomson, Uma S. Nair

**Affiliations:** 1 Public Health Department, Ministry of Public Health, Doha, Qatar; 2 Health Research Governance Department, Ministry of Public Health, Doha, Qatar; 3 Epidemiology and Biostatistics Department, Mel and Enid Zuckerman College of Public Health, University of Arizona, Tucson, Arizona, United States of America; 4 Family and Community Medicine, College of Medicine, University of Arizona, Tucson, Arizona, United States of America; Manipal Academy of Higher Education, INDIA

## Abstract

Smoking self-efficacy, described as confidence in one’s ability to abstain from smoking in high-risk situations is a key predictor in cessation outcomes; however, there is a dearth of research on factors that influence self-efficacy surrounding smoking behavior. This study examines factors associated with baseline self-efficacy among treatment seeking participants enrolled in a pilot feasibility smoking cessation study. Participants (n = 247) were daily male smokers, residents of Doha in Qatar (18–60 years) who were enrolled in a telephone-based smoking cessation study. Baseline assessments included self-efficacy, home smoking rules, socio-demographic variables, smoking history, and psychosocial characteristics. Factors associated with self-efficacy were assessed using multiple linear regression analysis. Results showed that after controlling for relevant variables, number of cigarettes smoked ($\hat{\beta }$ = -0.22; 95% CI: -0.37, -0.06), having at least one quit attempt in the past year ($\hat{\beta }$ = 2.30; 95% CI: 0.27, 4.35), and reporting a complete home smoking ban ($\hat{\beta }$ = 3.13; 95% CI: 0.56, 5.70) were significantly associated with higher self-efficacy to quit smoking. These results provide data-driven indication of several key variables that can be targeted to increase smoking self-efficacy in this understudied population.

## Introduction

Cigarette smoking remains the leading cause of many preventable diseases, with the number of smoking-attributable deaths expected to rise to 10 million per year by the year 2030 [1]. Smoking prevalence in the Eastern Mediterranean Region countries continues to be high unlike the decline observed in the rest of the world [2, 3]. Qatar is one of the countries in the region with high smoking rates, currently at 25.2% smoking prevalence among adults. This has led to country -wide efforts to implement strategies to reduce smoking prevalence [4] that has included nationwide comprehensive policies (e.g., taxation, smoke-free laws) with recent efforts to develop local quitlines to provide individual counseling, given this approach has been highly effective in promoting smoking cessation internationally [5]. A major component of the effectiveness of the behavioral approaches to smoking behavior change, is the building of client self-efficacy [6–9].

Self-efficacy refers to an individual’s confidence in their ability to perform a behavior [10] and has been considered an important component in behavior change theories with particular relevance in the area of smoking behavior change [11, 12]. Self-efficacy to abstain from smoking has been identified as a significant mediator/ predictor of treatment effectiveness in both pharmacological and behavioral smoking cessation intervention programs [6, 13–16]. Self-efficacy has been found to mediate the relationship between craving and tobacco abstinence among cardiac patients [17] and recent evidence revealed that this relationship was mediated by risk perception [18]. The dynamic association between self-efficacy and nicotine withdrawal (a significant predictor of smoking cessation and relapse) was examined by Morrell et al., who found that self-efficacy predicted severity of withdrawal during abstinence [19]. Self-efficacy is also associated with successful abstinence and relapse outcomes [6, 20] with increased abstinence self-efficacy being directly related to successful cessation outcomes [21]. Researchers have observed a bi-directional association between self-efficacy and smoking behavior [22] with self-efficacy predicting smoking abstinence as much as abstinence predicted self-efficacy. This finding is in line with behavioral theory and clinical research, which suggest that self-efficacy reflects, rather than causes, behavior change [6].

While the importance of self-efficacy has been commonly reported in the context of smoking cessation success little is known on factors that could be related to self-efficacy. Improving knowledge on the factors associated with baseline smoking self–efficacy at the time of enrolment in a quit smoking program can lead to efforts to tailor counseling strategies [14] that may perhaps address self-efficacy during a quit attempt. Given this gap, in the present study we sought to determine the factors that may be associated with baseline smoking cessation self-efficacy, among residents of Qatar who intend to quit smoking.

## Materials and methods

### Participants and design

The study sample included cessation treatment seeking male smokers, who were residents of Doha in Qatar, enrolled in a smoking cessation study. Details of the study protocol are described elsewhere [23]. Briefly, participants were recruited from the primary health care clinics (PHCCs) and based on the healthcare system, patients interested in quitting were referred to their closest smoking cessation clinic. The smoking clinic physician examined the patients, prescribed the appropriate smoking cessation medication (e.g., Champix or Zyban) and/or nicotine-replacement therapy (e.g., nicotine gum, patch, or lozenge) and referred participants to the study staff. To be eligible for the study, participants needed to be 18–60 years old, male, and daily smokers (smoking at least one cigarette per day for the past 7 days). Interested and eligible participants met with the study staff to complete informed consent, baseline assessments in Arabic or English, receive an orientation of the program and schedule of the counseling calls. The first call was initiated approximately 24–48 hours post-baseline where participants spoke with an assigned quit counselor. As a result of the COVID-19 outbreak recruitment process was shifted to remote, and participants provided audio recorded verbal consent. This study was reviewed and approved by the primary health care clinics (PHCCs) Institutional Review Board (IRB) in Qatar. Emergent changes to study protocols and consenting process post COVID were also approved by the PHCC IRB.

### Measures

All measures were self-reported and recorded at the time of enrollment (a single time point).

#### Primary outcome

Smoking self-efficacy was assessed by a 12-item questionnaire (SEQ-12), that measured confidence in the participant’s ability to refrain from smoking when faced with internal stimuli (e.g., feeling nervous, depressed) and external stimuli (e.g., being with other smokers). The questionnaire was translated into Arabic by a bilingual translator (native Arabic speaker) and was examined by two research team members fluent in Arabic for cultural competency. The scale has shown high internal consistency and is a valid and reliable scale for measuring self-efficacy [24]. The score for each question ranges on a 4-point Likert scale from 0–3. SEQ-12 score ranges from 0–36, with higher values indicating higher self-efficacy for smoking cessation. The aggregate score was only computed if no more than 2 out of the 12 questionnaire items had missing or ‘not applicable’ responses.

#### Independent variables

Our independent variables included: (a) *Home smoking rules* assessed by asking the participants to choose one of the statements that best describes their family’s rules about smoking inside the house. Response options included: "anyone can smoke anywhere and anytime at home", "smoking is allowed in some places and during certain times inside the home", "smoking is only allowed inside the home on special occasions or if a special guest is visiting", "smoking is not allowed anywhere inside the home". The responses were then categorized for analysis into "no ban" (anyone can smoke anywhere and anytime at home), "some ban" (smoking is allowed in some places and during certain times inside the home or smoking is only allowed inside the home on special occasions or if a special guest is visiting) and "complete ban" (smoking is not allowed anywhere inside the home); (b) *Depressive symptoms* assessed using the short-form of the Center for Epidemiological Survey for Depression (CESD) [25] consisting of 10 items to assess symptoms experienced in the past 7 days; (c) *Nicotine dependence* assessed using the Fagerström test for nicotine dependence (FTND) [26]. FTND is extensively used to measure the level of nicotine dependence and has good internal consistency and high test-retest reliability [27]; (d) *Smoking urge coping* assessed using a 12-item scale [28] to evaluate the use of cognitive and behavioral strategies for managing smoking urges. The questionnaire consisted of different strategies and the participant was asked how often in the last week he used any of these strategies to manage their urges to smoke from a scale of 1–4 (1 = never, 2 = rarely, 3 = sometimes, 4 = often).

#### Covariates

Covariates used in our analysis were identified from the literature, and included demographic measures; age (years), marital status (married or living with partner, currently not married or living with partner), education level (less than college degree, college degree and above) and employment status (employed, not employed). Other covariates also included number of years of regular cigarette smoking, number of daily cigarettes smoked (calculated by averaging over 7 days of a smoking timeline follow-back), number of smoking quit attempts over the last year (none, 1, ≥ 2), and e-cigarette use over the past 30 days (yes, no).

### Statistical analysis

Summary statistics (mean ± S.D. or frequency (%) were calculated for demographic, smoking and psychosocial characteristics at baseline. Bivariate analysis focused on identifying covariates that showed at least a marginally significant (p-value ≤ 0.10) association with SEQ-12 score. Pairwise correlations were computed for the following continuous variables: age at recruitment, number of years of regular cigarette smoking, average number of daily cigarettes smoked, FTND score, CESD-10 score, smoking urge coping score and SEQ-12 score. After assessment of normality for SEQ-12 score within each category, independent sample t-tests/one-way ANOVA or Mann-Whitney test/Kruskal Wallis test (as appropriate) were used to assess for significant difference of SEQ-12 score between categories of the following variables: marital status, education level, employment status, e-cigarette use over past 30 days, number of smokers in house beside themselves, number of smoking quit attempts over the last year, smoking rules within house and presence of a chronic condition. Factors associated with SEQ-12 score were assessed using multiple linear regression. Covariates were included based on literature and significant correlation/association with SEQ-12 score based on bivariate analysis. Change in multiple R^2^ values (by excluding a covariate from final model) was used to compare the relative importance of covariates as predictors of SEQ-12 score. The observations were assumed to be independent. Scatterplots were used to assess the linear relationship between the outcome and any continuous covariates, and any transformations were made if necessary. Residual histograms and residual vs fit plots were used to assess normality of residuals and homoscedasticity, respectively. Multiple imputation was carried out using the Markov-Chain Monte Carlo method to generate 10 imputed datasets (each with n = 233). Imputed variables included SEQ-12, CESD-10, FTND and smoking urge coping scores. Estimates from multiple linear regression models on imputed datasets were combined and compared to the original model to evaluate the robustness of findings. All analysis were performed using SAS 9.4 (SAS Institute Inc.).

## Results and discussion

Baseline characteristics for n = 247 male participants are presented in Table 1. The majority were of Egyptian nationality (29.6%) followed by Jordanians (1.8%) and Qataris (12.6%). On average, participants were 38.5 (±9.0) years of age, 81.7% reported being married and 93.5% were currently employed. Most of them reported smoking daily (97.9%) and on an average smoked 16.9 cigarettes per day. Almost half of the participants (45%) reported a complete home smoking ban, 30.6% had some bans and a quarter of the sample reported that smoking was allowed anywhere in the home.

**Table 1:** Baseline characteristics of participants (n = 247) presented as mean (±SD) or frequency (%).

Characteristics	% or Mean± SD
Age at recruitment, (years)	38.5±9.0
Nationality, n (%)	
Qatari	31 (12.6)
Egyptian	73 (29.6)
Jordanian	34 (13.8)
Syrian	23 (9.3)
Other	63 (25.5)
Missing	23 (9.3)
Marital Status^a^, n (%)	
Married or living with partner	201 (81.7)
Currently not married or living with partner	45 (18.3)
Education level^a^, n (%)	
Less than college degree	83 (33.6)
College degree and above	164 (66.4)
Employment status^a^, n (%)	
Employed	230 (93.5)
Not employed	16 (6.5)
Number of years of regular cigarette smoking	18.4 ± 8.4
Number of daily smoked cigarettes	16.9 ± 11.0
Number of smoking quit attempts over last year, n (%)	
None	149 (61.6)
1	56 (23.1)
≥ 2	37 (15.3)
E-cigarette use in the past 30 days^b^, n (%)	
Yes	44 (17.9)
No	197 (80.1)
Smokers in household beside yourself^a^, n (%)	
Yes	52 (21.1)
No	194 (78.9)
House smoking rules^b^, n (%)	
No ban	59 (24.4)
Some ban	74 (30.6)
Complete ban	109 (45.0)
FTND score	4.5± 1.0
CESD-10 score	7.3± 4.7
Smoking Urge Coping score	11.1± 7.9
SEQ-12 score	11.8± 7.5
Have at least one underlying chronic condition, n (%)	79 (32.1)

Abbreviations: CESD-10, Center for Epidemiological Studies Depression score; SEQ-12, Smoking Self-Efficacy Questionnaire Score; FTND, Fagerström score of nicotine dependence.

a. Individual (n = 1) responded ‘Don’t know’/ ‘Refused to answer’.

b. Individuals (n = 5) responded ‘Don’t know’/ ‘Refused to answer’.

Variables that showed significant bivariate associations with SEQ-12 score included average number of daily smoked cigarettes, smoking house rules, use of e-cigarette over last 30 days, at least one smoking quit attempt over the last year, nicotine dependence, and urge coping. Age, education, and depressive symptoms (CESD-10) were also included in the model based on literature. A total of n = 194 participants were included in the multiple regression model (Table 2). Estimates from the primary analysis showed that average number of daily smoked cigarettes, at least one smoking attempt over last year and a complete ban of smoking within house (compared to no ban) were significant predictors of smoking self-efficacy. Average number of daily smoked cigarettes and smoking house rules were also among the top three variables that accounted for the largest variation in SEQ-12 score.

**Table 2:** Parameter estimates from multivariable linear regression for SEQ-12 score and change in multiple R^2^(%) by exclusion from final model.

Variables	$\hat{\beta }$ (95% CI)	ΔR^2^(%)	Sensitivity
			$\hat{\beta }$ (95% CI)
	(n = 194)		
			(n = 233)
Age (years)	-0.02 (-0.14, 0.09)	0.44	-0.05 (-0.16, 0.07)
College Education or above^a^	0.17 (-1.96, 2.30)	0.23	0.74 (-1.26, 2.75)
FTND	1.44 (-0.28, 3.17)	18.12	1.64 (0.04, 3.23)
CESD-10	-0.15 (-0.38, 0.07)	31.72	-0.19 (-0.41, 0.03)
Smoking Urge Coping score	0.12 (-0.02, 0.25)	1.23	0.14 (0.01,0.28)
Average no. of daily smoked cigarettes	-0.22 (-0.37, -0.06)**	28.17	-0.23 (-0.38, -0.08)
Used e-cigarette over last 30 days	1.66 (-1.00, 4.31)	4.72	2.15 (-0.45,4.75)
At least 1 smoking quit attempt over last year^b^	2.30 (0.27, 4.35)*	15.60	1.93 (-0.01, 3.88)
Smoking house rules^c^		22.08	
Some ban	0.71 (-1.92, 3.34)		0.19 (-0.18, 4.84)
Complete ban	3.13 (0.56, 5.70)*		2.33 (-0.18, 4.84)

Abbreviations: St., Standardize; FTND, Fagerström score for nicotine dependence; CESD-10, Center for Epidemiological Studies Depression score.

Reference = “Below college level”.

Reference = “No quit attempt”.

Reference = “No ban”.

*p-value< 0.05

**p-value< 0.01.

## Discussion

To our knowledge, this is the first study examining factors related to self-efficacy in a population of adult males, residents of Qatar who are daily users of cigarettes. After controlling for relevant variables, results indicated that, number of cigarettes smoked, having at least one quit attempt in the past year, and having a complete home smoking ban, were associated with higher self-reported self-efficacy. While extent evidence indicates the mediating role of self-efficacy on successful smoking cessation outcomes across different populations [15, 17, 29], little is known on what factors are associated with smoking self-efficacy.

Higher number of cigarettes smoked at baseline was associated with lower baseline smoking self-efficacy in our sample. Ours was a population of relatively heavy smokers (average of 17 cigarettes per day) who had been smoking for an average of almost 20 years. While, compared to light and moderate smokers, heavy smokers are more likely to seek assistance to quit, they are less likely to be successful in maintaining long-term abstinence [30, 31]. Our findings suggest that smoking self-efficacy at baseline may be a potential mechanism that explains poor cessation outcomes among heavy smokers. Indeed, in a qualitative study assessing attitudes among heavy smokers, Thompson, et al. found that heavy smokers reported finding it extremely difficult to maintain abstinence for long periods of time, procrastinated in quitting, reinforced smoking, and experienced physical and psychological addictions to smoke [32]. Furthermore, in other studies, relapse at 1 and 3 months after a quit attempt was predicted by low levels of self-efficacy [14], and low self-efficacy was associated with an increase in nicotine withdrawal severity [19].

In our study, compared to having no or some smoking bans in the home, participants with a complete ban in the household were significantly more likely to have higher self-efficacy to refrain from smoking. Not only does having a smoke-free home significantly decrease exposure to second hand tobacco smoke to members in the household [33], but studies show significant associations between smoke-free homes and reduced cigarette consumption [34], increased quit attempts [35], increased quit duration, reduced relapse in cross-sectional and longitudinal samples [36], and healthier lifestyle, which, in turn, is correlated negatively to psychological distress and positively to psychological well-being [37]. Similar results are also seen among quitline callers, where implementing a home smoking ban significantly increased the likelihood of cessation among tobacco users utilizing quitline services [38]. Together, evidence may point to self-efficacy as a potential mediator between home smoking bans and behavior change. Smoke-free policies at home may facilitate a feeling of confidence that one can take control of a potentially harmful part of one’s life. In a similar context, investigating smoking bans at the workplace, a review found that smokers who work in companies with smoking bans, smoke less cigarettes daily, are more likely to be considering quitting, and a larger proportion of them quit, compared to employees at workplaces with no smoking bans [39]. These results provide more impetus for quitlines to incorporate strategies to help callers to create smoke-free zones in the home as part of increasing smoking cessation self-efficacy.

Next, compared to those with no quit attempts, participants reporting at least one quit attempt in the past year were more likely to report greater self-efficacy. Nicotine dependence is a chronic relapsing disorder with studies indicating that it may take someone thirty or more quit attempts before being successful [40]. On one hand, committing to quit may be difficult for smokers with prior quit attempts due to fear of failure and embarrassment if unsuccessful [41]. Alternately, engaging in a quit attempt allows a person to practice coping strategies and in turn increase self-efficacy to quit in the future with researchers recommending engaging smokers in practice quit attempts to promote cessation-like activities in a non-threatening manner [42, 43]. While we did not assess for specific strategies utilized during previous quit attempts (e.g., coping skills, pharmacotherapy, cold turkey) or reasons for quitting, it appears that similar factors may be accounting for the relationships between quit attempts and self-efficacy in our sample. Future studies in this population can engage smokers in practicing quit attempts prior to the goal of long-term cessation as part of the behavioral counseling intervention.

Though it contributed to the largest variance in the model, depression was not found to be related to self-efficacy in our study population. Available evidence suggests that depressive symptoms have been associated with low self-efficacy [44] but at the same time self-efficacy is likely to mediate the association between depression and smoking behavior [29]. The relationship between depressive symptoms and smoking self-efficacy is complex and future studies in this area could examine the potential mediators and moderators that influence the relationship between depression and smoking self-efficacy.

This study has some limitations that merit consideration. Our simultaneous measurements of the outcome variable and the potential factors do not allow for causal relationships to be inferred. To further examine how these factors may be associated with self-efficacy, it is necessary to follow up longitudinally with the study sample over a longer period. Second, although we were able to control for multiple covariates, this study included only adult male smokers in Qatar which restricts the generalizability of our findings as we were unable to examine for gender effects. However, this is the first study to provide information on smoking cessation predictors for a population in the Middle East area. Finally, the baseline assessments were self-reported and subjectivity and recall bias is possible to have affected our observations. Underreporting is also a possibility in self-reported measures. While the self-efficacy questionnaire was translated and tested for cultural relevance, it has not been validated. Future validation studies are needed to assess its suitability for this population.

## Conclusions

In summary, our findings provide insight into the factors that may be associated with higher or lower self-efficacy before the quit day, among adult male smokers, residents of Qatar who want to quit smoking. These observations support the importance of assessing and targeting factors that in turn may aid in increasing self-efficacy for participants enrolled in smoking cessation programs in the Middle East, a region with high tobacco burden.

## Supporting information

S1 File Data set with all the calculated scores. (XLSX)

S2 File Description and coding of the variables used. (XLSX)
